# Utilization of Calcium Carbide Residue Using Granulated Blast Furnace Slag

**DOI:** 10.3390/ma12213511

**Published:** 2019-10-26

**Authors:** Joonho Seo, Solmoi Park, Hyun No Yoon, Jeong Gook Jang, Seon Hyeok Kim, H. K. Lee

**Affiliations:** 1 Department of Civil and Environmental Engineering, Korea Advanced Institute of Science and Technology, 291 Daehak-ro, Yuseong-gu, Daejeon 34141, Korea; junhoo11@kaist.ac.kr (J.S.); solmoi.park@kaist.ac.kr (S.P.); yhn0307@kaist.ac.kr (H.N.Y.); kimsh1187@kaist.ac.kr (S.H.K.); 2 Division of Architecture and Urban Design, Institute of Urban Science, Incheon National University, 119 Academy-ro, Yeonsu-gu, Incheon 22012, Korea; jangjg@inu.ac.kr

**Keywords:** slag, calcium carbide residue, solidification, stabilization, cementitious material, characterization

## Abstract

The solidification and stabilization of calcium carbide residue (CCR) using granulated blast furnace slag was investigated in this study. CCR binding in hydrated slag was explored by X-ray diffraction, ^29^Si and ^27^Al magic angle spinning (MAS) nuclear magnetic resonance (NMR) spectroscopy, and thermodynamic calculations. Mercury intrusion porosimetry and and compressive strength tests assessed the microstructure and mechanical properties of the mixtures of slag and CCR. C-A-S-H gel, ettringite, hemicarbonate, and hydrotalcite were identified as the main phases in the mixture of slag and CCR. The maximum CCR uptake by slag and the highest volume of precipitated solid phases were reached when CCR loading in slag is 7.5% by mass of slag, according to the thermodynamic prediction. This feature is also experimentally observed in the microstructure, which showed an increase in the pore volume at higher CCR loading.

## 1. Introduction

Harmful wastes generated by the results of industrialization are rapidly increasing beyond the limit of a naturally purifiable level in qualitative and quantitative aspects [1,2]. These wastes must be safely disposed of through proper treatments so as to segregate them from living creatures. Organic wastes can be converted into innocuous substances by modifying the molecular structure of the wastes via incineration, while the dismantlement of inorganic wastes is far beyond simplicity except in some cases [3,4,5]. Therefore, the solidification and stabilization (SS) method has been widely utilized as measures to stably dispose the inorganic wastes in a harmless way [2,6]. The SS typically refers to a method of immobilizing wastes, either physically or chemically, alone or in combination, to fix the wastes in a mass solidifier or to cover the superficial layer of the wastes [2,6]. Thereby, the SS process facilitates handling, improves physical and chemical properties, and inhibits leaching of the wastes.

Calcium carbide residue (CCR) is an industrial waste generated during the manufacturing process of acetylene gas, and is formed as shown in Equation (1) [7].

CaC_2_ + 2H_2_O → C_2_H_2_ + Ca(OH)_2_ (1)

Since calcium hydroxide is a primary component of CCR, it forms an alkaline environment when dissolved in water. Due to its basic characteristic, CCR is mostly buried in a designated disposal area rather than being recycled [7]. Currently, the possible recycling strategy of CCR may include the use of CCR as a neutralizer of acidified soil. Nevertheless, the disposal of CCR poses edaphic threats, especially the basification of soil and subsurface water pollution due to alkaline contamination [8]. It was reported that approximately 21,500 ton of the CCR is generated in Thailand annually in a slurry form [9] and the CCR can hardly be utilized in any practical work [7]. In addition, the toxicity of the CCR (alkaline pH of the waste) can significantly influence the heterotrophic microorganisms whose feeding is heavily dependent on a detrital input [10]. As Equation (1) illustrates, 32 g of the calcium carbide yields 13 g of acetylene gas with 37 g of the CCR, which indicates that a relatively large amount of CCR is generated considering the amount of produced acetylene gas, which, in turn, is a significant environmental burden either from the preparation of the disposal area of from a basification of the surroundings. Therefore, proper SS of CCR is required. 

Several studies have been carried out to solidify/stabilize CCR by utilizing the basic characteristic of CCR when dissolved in water. These studies have revealed the possibility of utilizing CCR with siliceous materials that have pozzolanic properties, which can result in the formation of a gel with a binding or cementing property [8,11,12,13]. Makaratat et al. investigated the use of CCR with fly ash to solidify the CCR in a cementitious matrix [11]. In their study, CCR-fly ash concrete yielded a compressive strength and an elastic modulus of 28.4 MPa and 27.7 GPa, respectively, after 28 days of curing, which are similar values to those displayed in concrete made with ordinary Portland cement. Jaturapitakkul and Roongreung [8] assessed the potential of solidifying the CCR with rice husk ash, concluding that the sample with rice husk ash and CCR in a 50:50 ratio displayed the compressive strength up to 15.6 MPa after 28 days of curing. 

Although there have been some studies pertaining to the use of CCR with pozzolanic materials, the investigation of SS of CCR using slag, an industrial by-product of iron processing, has not yet been studied. It should be noted that the investigations regarding the characterization of the slag-portland tie system have been discussed. However, studies of utilization of CCR using slag is not yet available and, hence, can provide a fundamental understanding for the field. This study, therefore, provides an understanding of the chemistry of the mixtures of slag and CCR by investigating microstructural and mechanical characteristics of the material at various CCR/slag ratios. The reaction products and reaction degree of the mixtures of slag and CCR were explored by means of X-ray diffractometry (XRD, Rigaku, Tokyo, Japan), solid-state ^29^Si and ^27^Al magic angle spinning (MAS) nuclear magnetic resonance (NMR) spectroscopies (Bluker, Billerica, MA, USA), and thermodynamic modelling. Pore characteristics and corresponding mechanical strength were investigated by means of mercury intrusion porosimetry (MIP) and an unconfined compressive strength test, respectively. The outcomes of this study are highly anticipated to provide a fundamental knowledge in the S/S of the CCR by using slag. In addition, since the previous studies of the utilization of CCR with siliceous material require heat supplies during curing and show low mechanical strength, the present topic can genuinely be efficient for dealing with the generated CCR in an eco-friendly and engineering way.

## 2. Experimental Program

### 2.1. Materials and Sample Preparation

**Figure 1:**
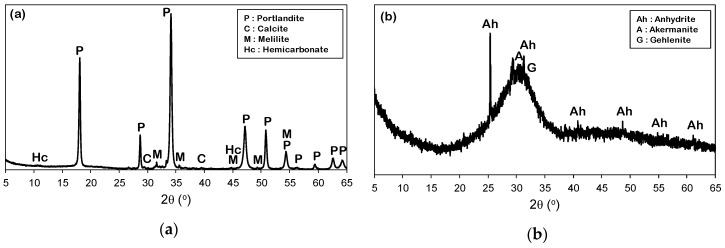
X-ray diffractograms of (**a**) CCR and (**b**) raw slag.

The chemical compositions and XRD patterns of the CCR (provided by Kyungin Eco Chemical Co., Ltd., Ansan, Korea) and the slag used in this study are provided in Table 1, and Figure 1, respectively. The CCR used in this study consists mainly of portlandite due to the presence of calcium hydroxide, which is confirmed by its XRD pattern that shows predominant peaks corresponding to portlandite (Ca(OH)_2_, PDF #00-044-1481). A minor quantity of other phases melilite glass (Na_1.99_Ca_1.99_Al_2_Si_4_O_14_, PDF #00-024-1063), calcite (CaCO_3_, PDF #01-072-1937), and hemicarbonate (Ca_4_Al_2_O_7_(CO_2_)_0.5_·12H_2_O, PDF #00-036-0129) was also identified. In the region of 17–38° (2θ), the slag showed an amorphous hump with notable peaks, which corresponds to gehlenite (Ca_2_Al_2_SiO_7_, PDF #00-035-0755), åkermanite (Ca_2_MgSi_2_O_7_, PDF #01-074-0990), and anhydrite (CaSO_4_, PDF #01-072-0916).

The mixtures of slag and CCR were made by increasing the amount of CCR in the binder (CCR + slag) from 10 to 40 wt. %, with an identical water-to-binder ratio of 0.5. Mortar specimens with identical CCR/binder ratios were manufactured, for the purpose of testing the compressive strength, with a mass ratio for water: binder: aggregate of 1:2:3 with river sand as an aggregate. The sample IDs have been designated according to the mass percentage of the CCR in the binder composition (i.e., C10 denotes the sample with 10 wt. % CCR). A mixture of ingredients was agitated for 5 min at room temperature (25 °C) and then cured in 50 mm—cubical molds. The samples were sealed with a plastic wrap and cured until the testing day.

### 2.2. Test Methods

The samples were characterized after 28 days of curing by various test methods. High-resolution XRD was performed using an Empyrean instrument with CuKα radiation at 40 kV and 30 mA. The XRD pattern of the samples were recorded in a range of 5–65° (2θ) with a step size and time per step of 0.026° (2θ) and 1.58 s, respectively. The International Center for Diffraction Data (ICDD) PDF database was referenced to identify crystalline phases. 

Solid-state ^27^Al MAS NMR spectra were obtained at 104.3 MHz using an HX CPMAS probe under ambient conditions. Pulse width and relaxation delay of 1.2 μs and 2 s were used, respectively. An external aqueous AlCl_3_ sample was used to calibrate the chemical shift at 0 ppm. Solid-state ^29^Si MAS NMR spectra were obtained at 79.51 MHz using an HX-CPMAS probe with a 4 mm outer diameter (o.d.) zirconia rotor under an ambient condition. Pulse width and a relaxation delay of 1.6 μs and 10 s were used, respectively. An external tetramethylsilane (TMS) was used to calibrate the chemical shift at 0 ppm. 

The uptake of CCR by slag was modelled using the Gibbs free energy software GEM-Selektor v.3.5 (http://gems.web.psi.ch/), and coupling with Cemdata18 [14]. The extended Debye–Hückel equation was used to estimate the activity coefficients of aqueous species [15]. The parameter for common short-range interactions, and the average ion size of charged species were 0.098 kg/mol and 3.31 Å, respectively (i.e., NaOH-dominated electrolytes). The phase composition of the system involving CCR, slag, and mixing water was simulated in the conditions identical to the experiments (25 °C and 1 bar).

The pore characteristics of samples were estimated using MIP. The MIP was performed using an AutoPore IV 9500 V.1.05 machine (Micromeritics Corp., Norcross, GA, USA). The pressure used in the MIP analysis ranged from 0.00069 to 414 MPa (from 1 to 60,000 psi). 

Unconfined compressive strength was assessed by a compression device at a constant descending rate of 0.02 mm/s. The average strength was determined from three replicas.

## 3. Results and Discussion

### 3.1. X-Ray Diffraction

**Figure 2:**
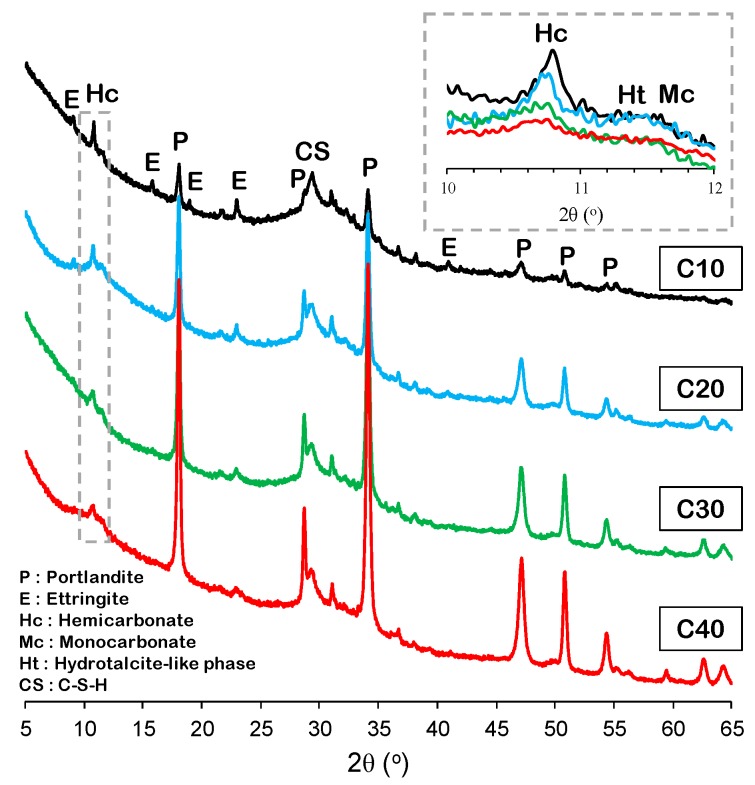
X-ray diffractograms of the mixtures of slag and CCR.

The XRD patterns of the mixtures of slag and CCR with a magnified angular region of 10–12° (2θ) are depicted in Figure 2. The main reaction product of the samples is identified as calcium-silicate-hydrate (C-S-H, Ca_1.5_SiO_3.5_H_2_O, PDF #00-033-0306), as depicted in the XRD patterns in this study and reported in studies where binders with similar chemistries were studied [16]. Moreover, this phase is expected to have some degree of Al-substitution for Si at the bridging and paired sites [17]. Ettringite (Ca_6_Al_2_(SO_4_)_3_(OH)_12_·26H_2_O, PDF #00-013-0350) was commonly identifiable in all samples, while its intensity diminished as the CCR content increased. The peak intensity of portlandite increased as the amount of CCR increased. The samples commonly showed peaks corresponding to AFm-group minerals such as hemicarbonate (Ca_4_Al_2_O_7_(CO_2_)_0.5_·12H_2_O, PDF #00-036-0129), the hydrotalcite-like phase (Mg_4_Al_2_(OH)_12_CO_3_·3H_2_O, PDF #00-014-0525), and monocarbonate (Ca_4_Al_2_O_7_(CO_2_)·11H_2_O, PDF #00-036-0377). The presence of Afm phases with carbon dioxide as an interlayer species (i.e., hemicarbonate and monocarbonate) can be attributed to the presence of calcite in the CCR. Consequently, the phases that are responsible for binding CCR, including C-S-H and Aft/Afm phases, do not alter greatly during greater CCR-loading. 

### 3.2. ^27^Al MAS NMR

**Figure 3:**
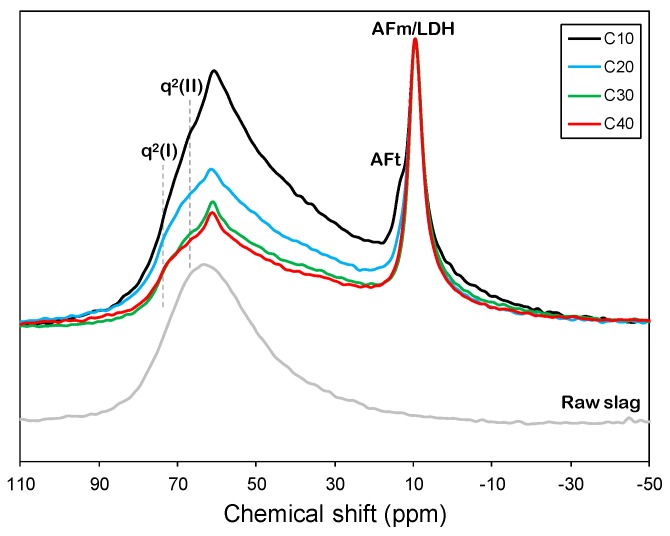
Normalized ^27^Al MAS NMR spectra of raw slag and mixtures of slag and CCR.

The normalized ^27^Al MAS NMR spectra of the raw slag and the mixtures of slag and CCR are shown in Figure 3. The raw slag displayed a hump between 80 and 30 ppm, which ranged throughout tetrahedral and pentahedral Al [18].

The samples showed resonance between 80 and 50 ppm, which corresponds to the silicate chain of the C-A-S-H incorporating tetrahedrally coordinated Al [19]. In addition, all samples showed resonance at 10 ppm corresponding to octahedral Al, which is attributed to the AFm phase [20]. The intensity of the signal originating from the tetrahedral Al (i.e., q^2^(I) and q^2^(II)) featured in the C10 sample was significantly higher than in the other samples. This indicates that the C-A-S-H contained a significant amount of Al [21]. It should, however, be noted that this observation might be influenced by the slag/CCR ratio. In particular, the relative resonance intensity at 68 ppm, corresponding to q^2^(II), showed a noticeable difference between samples, which implies that the extent of cross-linked Al in the C-A-S-H decreased as the CCR content increased [22,23,24,25]. Additionally, a weak resonance at around 13 ppm, corresponding to AFt, gradually emerged as the CCR content decreased, which is in good agreement with the XRD patterns [26]. This observation can be explained by the remainder of sulfate or carbonate ions after forming hemicarbonate or monocarbonate. As revealed by the XRD patterns, sulfate ions in the monosulfate were substituted by carbon dioxide. The presence of carbon dioxide can be attributed to the nature of the raw material, which yields free sulfate ions. Consequently, unconverted monosulfate with a supply of free sulfate ions then led to the formation of ettringite [27]. Thus, the amount of ettringite present was proportional to the amount of hemicarbonate, as shown in the XRD patterns.

### 3.3. ^29^Si MAS NMR

**Figure 4:**
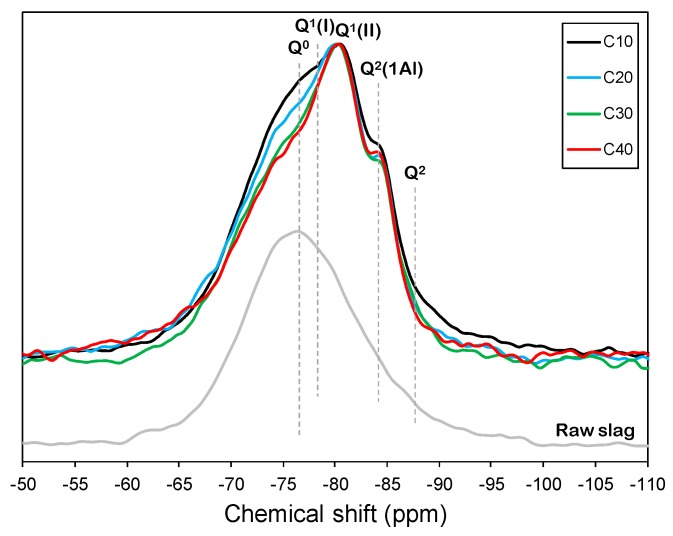
Normalized ^29^Si MAS NMR spectra of raw slag and mixtures of slag and CCR.

The normalized ^29^Si MAS NMR spectra of the raw slag and the mixtures of slag and CCR are shown in Figure 4. The deconvolution results and estimated mean chain length (MCL) of the individual spectrum are displayed in Table 2. The raw slag showed a wide resonance centered at around −75 ppm. 

The samples showed resonances at around −78 and −81 ppm, where each corresponded to the Q^1^ site charge-balanced with monovalent and divalent cations, respectively. In addition, the samples showed resonances at around −84 and −87 ppm, which corresponded to Q^2^(1Al) and Q^2^ sites, while the unreacted slag gave rise to the Q^0^ site [17,28]. The mean chain length (MCL) was estimated according to the relative fractions of the Q^n^, as expressed in Equation (2) [29]. The C-A-S-H in the C10 sample was found to have the longest MCL, while the MCL values of the samples with > 10 wt. % CCR were all similar.

MCL = 2(Q^1^ + Q^2^ + 1.5Q^2^(1Al))/Q^1^ (2)

The relative area corresponding to the anhydrous slag appears to decrease as the CCR content increases, which means that the reaction degree of slag was enhanced after 28 days of curing as the CCR content increased. The degrees of the slag’s reaction after 28 days were 49.2% and 57.4%, respectively, for the C10 and C40 samples.

### 3.4. Thermodynamic Modeling

**Figure 5:**
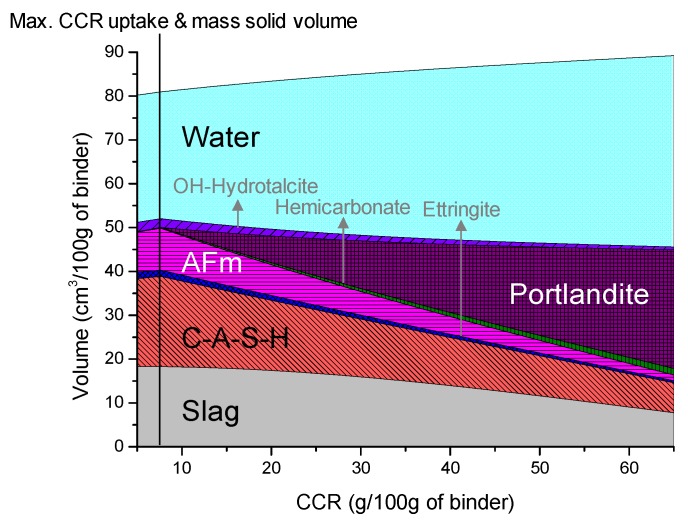
Calculated phase composition of the mixtures of slag and CCR.

The calculated phase composition of the mixtures of slag and CCR is shown in Figure 5. The reaction products of CCR and slag mixed with water are predicted by thermodynamic modeling, using the reaction degree of the slag obtained by the deconvolution process of the ^29^Si MAS NMR spectra [30,31]. The obtained reaction degree was used to extrapolate the result to 5 and 65 g of CCR per 100 g of the binder. The thermodynamic calculation of the system in Figure 5 predicts a phase composition similar to the XRD patterns, which shows that C-A-S-H, ettringite, hemicarbonate, hydrotalcite, and portlandite precipitate in a wide range of CCR/binder. A particular discrepancy between the thermodynamic calculation and the XRD patterns is that AFm-SO_4_/OH solid-solutions are identified as a major phase in the calculation, which could not be detected by X-ray due to the amorphous nature [32]. Hence, the decreased intensity of hemicarbonate in the XRD pattern can be due to the reduced amount of the overall AFm-related phases, as suggested by the calculation.

The volume of the reaction products formed trends to decrease since the solid volume is being gradually occupied by portlandite (i.e., residual CCR). The amount of CCR that leads to the highest amount of solid phase precipitation is predicted as 7.5 g per 100 g of binder, and coincides with the precipitation of portlandite and greater amount of residual water. Hence, the pore volume of the system is likely to increase with the increasing amount of CCR in the binder composition.

### 3.5. Pore Characteristics

**Figure 6:**
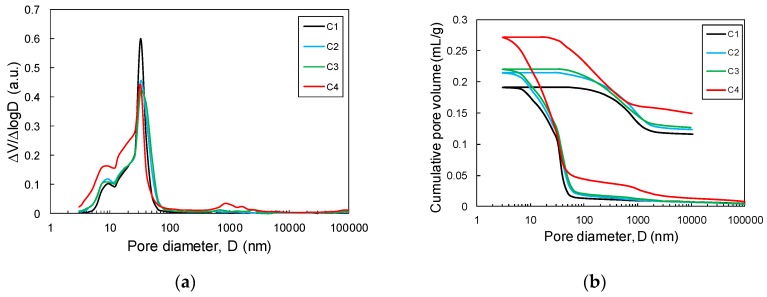
Mercury intrusion porosimetry test results of the mixtures of slag and CCR: (**a**) log differential intrusion curve and (**b**) cumulative intrusion curve.

The pore characteristics of the mixtures of slag and CCR, as measured by the MIP test, are shown in Figure 6 and tabulated in Table 3. The log differential curve of the mixture of slag and CCR showed a significant development of pores that have diameters below 80 nm. The C10 sample showed a noticeable amount of pores with dimeters ranging from 10 to 100 nm, which is mainly attributed to the presence of C-A-S-H [33]. This observation is in fair agreement with the thermodynamic calculation, which predicted the amount of C-A-S-H to decrease with an increase of the CCR input. Furthermore, the C40 sample showed a considerable amount of pores with a diameter of 1000 nm, which is attributed to the inclusion of entrained air [34]. In addition, the increase in the cumulative pore volume with an increase of the CCR content was closely related to the decreasing solid phase precipitation and the greater amount of residual water, as predicted in the calculated phase composition.

### 3.6. Unconfined Compressive Strength

**Figure 7:**
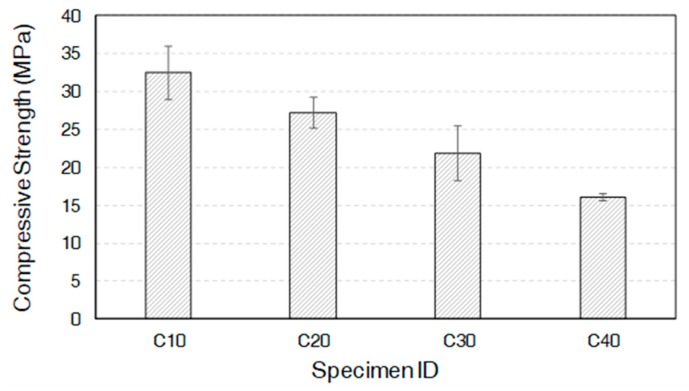
Unconfined compressive strength of the mixtures of slag and CCR.

The unconfined compressive strength of the mixtures of slag and CCR is shown in Figure 7. It can be clearly seen that the compressive strength of the mixtures of slag and CCR linearly decreased as the CCR content increased. This indicates that strength development is highly dependent on the amount of slag in the binder, given a sufficient amount of CCR is present in the system. An increase in the amount of CCR, which would decrease the overall amount of slag in the binder composition, may reduce the compressive strength. The unconfined compressive strength of the mixture of slag and CCR revealed that a mixture with the appropriate proportions could be designed to achieve desirable mechanical strength (up to 32.4 MPa). Higher doses of CCR (i.e., the C20, C30, and C40 samples) may be less effective in terms of strength development. The compressive strength of slag activated by pure CaO showed 42 MPa after 28 days of curing [16]. This is due to the linear increase in the porosity and the decrease in the amount of C-A-S-H when the CCR content increases. These are key strength-giving factors [35].

## 4. Conclusions

The present study investigated CCR binding in a hydrated blast furnace slag using XRD and solid-state MAS NMR spectroscopy. Characterization of the mixtures of slag and CCR after 28 days of reaction revealed that the main CCR-binding phases of the hydrated slag are C-A-S-H, ettringite, hemicarbonate, and hydrotalcite. The degree of reaction of the slag increased at higher CCR loading.

The predicted phase composition with calculations based on the reaction degree of the slag obtained by the spectral deconvolution of the ^29^Si MAS NMR spectra showed similar findings. Furthermore, the thermodynamic calculation predicted that the maximum CCR loading and the highest volume of precipitated solid phases occur at approximately 7.5 g of CCR per 100 g of slag. 

Both thermodynamic calculation and MIP indicated an increase in the pore volume in the binding matrix when a higher volume of CCR is incorporated. The increased pore volume at higher CCR loading is, thus, responsible for the decreased compressive strength of the samples incorporating a higher amount of CCR.

## Figures and Tables

**Table 1:** Chemical compositions of the CCR and slag used in this study.

(wt.%)	Al_2_O_3_	CaO	Fe_2_O_3_	MgO	Na_2_O	P_2_O_5_	SiO_2_	TiO_2_	LOI ^1^
CCR	1.83	70.10	0.14	0.50	0.05	0.01	4.70	0.02	21.60
Slag	11.50	47.70	0.60	3.00	-	0.60	32.40	0.50	0.29

^1^ Loss-on-ignition.

**Table 2:** Deconvolution results of ^29^Si MAS NMR spectra of CCR-loaded slag. The estimated chi-square tolerance value is 10^−6^. The provided data are the chemical shift (ppm) and integration of the relative area (%).

Specimen ID	Anhydrous Slag		Q^1^				Q^2^(1Al)		Q^2^		Q^3^(1Al)		MCL
	ppm	%	ppm	%	ppm	%	ppm	%	ppm	%	ppm	%	
C10	−74.0	50.8	−78.5	16.8	−80.8	9.0	−84.0	16.5	−87.0	6.5	−89.0	0.4	4.42
C20	−74.0	46.6	−77.0	10.9	−80.9	31.2	−84.7	7.1	−86.7	4.3			2.72
C30	−74.0	44.0	−78.5	19.5	−81.0	22.3	−84.5	5.1	−86.0	9.1			2.80
C40	−74.0	42.6	−78.7	22.5	−80.9	21.2	−84.6	10.6	−86.9	3.0			2.87

**Table 3:** Pore characteristics of mixture of slag and CCR as measured by MIP.

Specimen ID	Median Pore Diameter (nm)	Porosity (%)	Total Intrusion Volume (mL/g)
C10	17.9	35.5	0.19
C20	14.2	38.0	0.21
C30	14.4	38.7	0.22
C40	10.0	42.9	0.27
